# A randomized controlled trial testing a hyaluronic acid spacer injection for skin toxicity reduction of brachytherapy accelerated partial breast irradiation (APBI): a study protocol

**DOI:** 10.1186/s13063-018-3035-3

**Published:** 2018-12-17

**Authors:** Gerson M. Struik, Jeremy Godart, Gerda M. Verduijn, Inger-Karine Kolkman-Deurloo, Kim C. de Vries, Raymond de Boer, Linetta B. Koppert, Erwin Birnie, Ali Ghandi, Taco M. Klem, Jean-Philippe Pignol

**Affiliations:** 1 Department of Surgery, Franciscus Gasthuis and Vlietland, PO Box 10900, 3004 BA Rotterdam, The Netherlands; 2 Department of Radiation Oncology, Erasmus MC Cancer Institute, PO Box 5201, 3008 AE Rotterdam, The Netherlands; 3 Department of Surgery, Erasmus MC Cancer Institute, PO Box 5201, 3008 AE Rotterdam, The Netherlands; 4 Department of Statistics and Education, Franciscus Gasthuis and Vlietland, PO Box 10900, 3004 BA Rotterdam, the Netherlands; 5 Department of Genetics, UMC Groningen, University of Groningen, P.O. Box 30001, 9700 RB Groningen, the Netherlands; 6 Department of Radiology, Franciscus Gasthuis and Vlietland, PO Box 10900, 3004 BA Rotterdam, the Netherlands; 7 Department of Radiation Oncology, Dalhousie University, 5820 University Avenue, Halifax, NS B3H1V7 Canada

**Keywords:** Breast neoplasms, Partial breast irradiation, Brachytherapy, Permanent breast seeds implant, Skin toxicity reduction, Telangiectasia, Spacer, Hyaluronic acid

## Abstract

**Background:**

Accelerated partial breast irradiation (APBI) is a treatment option for selected early stage breast cancer patients. Some APBI techniques lead to skin toxicity with the skin dose as main risk factor. We hypothesize that a spacer injected between the skin and target volume reduces the skin dose and subsequent toxicity in permanent breast seed implant (PBSI) patients.

**Methods:**

In this parallel-group, single-center, randomized controlled trial, the effect of a subcutaneous spacer injection on skin toxicity among patients treated with PBSI is tested. Eligibility for participation is derived from international guidelines for suitable patients for partial breast radiotherapy, e.g. women aged ≥ 50 years with a histologically proven non-lobular breast carcinoma and/or ductal carcinoma in situ (DCIS), tumor size ≤ 3 cm, node-negative, and PBSI technically feasible. Among exclusion criteria are neoadjuvant chemotherapy, lymphovascular invasion, and allergy for hyaluronic acid. For the patients allocated to receive spacer, after the PBSI procedure, 4–10 cc of biodegradable hyaluronic acid (Barrigel™, Palette Life Sciences, Santa Barbara, CA, USA or Restylane SubQ®, Galderma Benelux, Breda, the Netherlands) is injected directly under the skin using ultrasound guidance to create an extra 0.5–1 cm space between the treatment volume and the skin. The primary outcome is the rate of telangiectasia at two years, blindly assessed using Bentzen’s 4-point scale. Secondary outcomes include: local recurrence; disease-free and overall survival rates; adverse events (pain, redness, skin/subcutaneous induration, radiation dermatitis, pigmentation, surgical site infection); skin dose; cosmetic and functional results; and health-related quality of life.

A Fisher’s exact test will be used to test differences between groups on the primary outcome.

Previous studies found 22.4% telangiectasia at two years. We expect the use of a spacer could reduce the occurrence of telangiectasia to 7.7%. A sample size of 230 patients will allow for a 10% lost to follow-up rate.

**Discussion:**

In this study, the effect of a subcutaneous spacer injection on the skin dose, late skin toxicity, and cosmetic outcome is tested in patients treated with PBSI in the setting of breast-conserving therapy. Our results will be relevant for most forms of breast brachytherapy as well as robotic radiosurgery, as skin spacers could protect the skin with these other techniques.

**Trial registration:**

Netherlands Trial Register, NTR6549. Registered on 27 June 2017.

**Electronic supplementary material:**

The online version of this article (10.1186/s13063-018-3035-3) contains supplementary material, which is available to authorized users.

## Background

Breast cancer is increasingly diagnosed at an early stage [1, 2]; for that stage, breast-conserving therapy, which includes wide local excision and radiotherapy, is equivalent to mastectomy in terms of local control and overall survival [3, 4]. These oncological outcomes are excellent in early-stage breast cancer patients [2]. Hence, radiotherapy essentially provides a cosmetic and quality-of-life benefit over mastectomy [5]. Since local recurrences usually occur close to the primary tumor [6], the concept of accelerated partial breast irradiation (APBI) was introduced [7] to both reduce the amount of breast tissue irradiated and enable faster treatment. For well-selected patients, APBI has been tested and validated through large randomized clinical trials (RCT), using either brachytherapy [8–11], external 3D conformal radiotherapy [12, 13], or intraoperative radiotherapy [14, 15].

Brachytherapy has been the most evaluated technique and recent advances beyond multicatheter implantation include balloon or strut brachytherapy as well as permanent breast seed implants (PBSI) [16, 17]. Brachytherapy is generally well tolerated and reported long-term toxicities are acceptable. A lower incidence of low-grade acute skin toxicity for APBI, 21% vs 86% for whole breast radiotherapy (*p* < 0.001) has been reported for the GEC-ESTRO trial [18]. Regarding late side effects at five-year follow-up, lower rates of severe grade 2–3 skin, 6.9% vs 10.7%, and similar rates of subcutaneous side effects, 12.0% vs 9.7%, were found in this study [19]. On the other hand, in a retrospective analysis of 1034 breast patients treated at The Ohio State University including 31% treated with a balloon applicator, Wobb reported more seroma grade 2 or higher (14.4% vs 2.9%, *p* < 0.001), more painful fat necrosis (10.2% vs 3.6%, *p* < 0.001), and more telangiectasia grade 2 or higher (12.3% vs 2.1%, *p* < 0.001) for APBI compared to whole breast radiotherapy [20]. Among those permanent side effects, increased painful seroma is almost exclusively due to balloon applicator, fat-necrosis can be due to multiple factors, while telangiectasia is almost exclusively due to an excess of dose to the skin. This makes telangiectasia a specific marker of radiation toxicity [21, 22]. Telangiectasia corresponds to the dilation of an abnormal neo-vasculature in the skin following the destruction of normal capillaries by the radiation treatment, resulting in visible vessels [23]. Although rates are lower than with whole breast irradiation, in breast brachytherapy 10–27% [9, 19, 24] of the patients develop some grade of telangiectasia. The majority of lesions are grade 1 (< 1 cm^2^) in breast radiotherapy studies reporting on late skin toxicity [9, 25, 26]. The onset of telangiectasia is from six months to 10 years after radiotherapy delivery [23]; however, rates of telangiectasia peak at two years with PBSI. Although permanent in most cases, some authors report disappearing of the telangiectasia with longer follow-up [9, 27]. Nevertheless, if present, telangiectasia can remind patients of their cancer similar to a surgical scar and have a direct negative impact on the cosmetic outcomes [9, 26].

Several authors recommend keeping a distance of at least 5 mm between the planning target volume (PTV) and the skin [28, 29] and limiting the maximum skin dose to 70% [8]. However, such constraints are not always achievable. A simple solution would be the use of a spacer material injected subcutaneously to move the skin out of the high dose region [21].

In this manuscript, we report the protocol of a RCT investigating the clinical benefit of a subcutaneous spacer injection on the skin dose, late skin toxicity, and cosmetic outcome in patients treated with low dose rate (LDR) seed brachytherapy. For this study, the breast skin is considered as a critical structure for the radiotherapy and the clinical outcomes are measured using a breakdown of traditional skin toxicity scales in order to capture the toxicity that is specific to radiotherapy [9, 21, 23, 30].

## Methods/design

### Aim and design

We propose a parallel-group RCT comparing the occurrence of telangiectasia at two years in PBSI patients with or without a subcutaneous spacer injection. Allocation ratio is 1:1 and the trial is designed to test the superiority of the intervention. The primary hypothesis for the trial assumes that an injected hyaluronic acid spacer will reduce skin dose of PBSI and eventually the rate of telangiectasia at two years, compared to patients undergoing PBSI without spacer. As the intervention is applied when the patient is sedated, a placebo injection as comparator was deemed unnecessary. The methods section is described according to the Standard Protocol Items: Recommendations for Interventional Trials (SPIRIT) 2013 checklist (see Additional file 1).

Eligible patients will be recruited at hospitals referring patients after breast-conserving surgery for adjuvant radiotherapy at the Erasmus MC Cancer Institute, a large University hospital in Rotterdam, where the PBSI technique can be performed in the Netherlands.

### Eligibility and exclusion criteria

Eligibility criteria were derived from international guidelines [31, 32] for suitable patients for partial breast radiotherapy. Eligible patients are females aged ≥ 50 years with a confirmed histological diagnosis of invasive ductal carcinoma (IDC) and/or papillary, tubular, cribriform or medullar carcinoma and/or ductal carcinoma in situ (DCIS), after breast-conserving surgery with axillary node dissection (with a minimum of six nodes sampled) or sentinel lymph node biopsy. The maximum dominant tumor size is 3 cm and the tumor must be excised with negative surgical margins at ink for invasive carcinoma and ≥ 2 mm negative margins for DCIS or have a negative re-excision. The PBSI should be deemed technically feasible based on the seroma location, visibility, and size performing an ultrasound; the total implanted volume should be < 150 cc. Patient should have signed an informed consent.

Ineligible patients include those with lymphovascular invasion, lobular features on histology (pure or mixed) or sarcoma histology, triple negative tumors, extensive in situ carcinoma, multicentric disease (in more than one quadrant or separated by ≥ 2 cm), bilateral breast cancer, recurrent breast cancer, Paget’s disease of the nipple, metastases or active other cancer (defined by any malignancy in < 5 years, excluding any cured non-melanoma skin cancer or cervical cancer), neoadjuvant chemotherapy, known allergy for hyaluronic acid, active auto immune disorder with severe vasculitis component, uncontrolled and complicated insulin-dependent diabetes, pregnancy, cosmetic breast implants, psychiatric or addictive disorder that would preclude attending follow-up, or postoperative wound infection or abscess following Centers for Disease Control and prevention (CDC) criteria.

### Interventions

The permanent seed implant procedure includes a computed tomography (CT) simulation done positioning the patient similarly to for external beam breast radiotherapy. The clinical target volume (CTV) corresponds to the seroma with a 1-cm margin, limited to the fascia pectoralis, and 5 mm below the skin; the planned target volume (PTV) is an additional expansion of 0.5 cm with the same skin and chest wall limits. A pre-implant plan is generated using the MIM Symphony® software (MIM Software Inc., Cleveland, OH, USA) to order stranded ^103^Pd seeds of 2.5 U activity.

For the procedure, anesthesia includes a non-steroidal anti-inflammatory drug (NSAID) for two days, light sedation with Propofol, and local freezing. Patients are positioned on a breast board, with the arm abducted similarly to the CT simulation. The breast skin is disinfected and the patient draped. The PTV projection perpendicular to the fiducial needle axis is outlined on the skin surface and verified using ultrasound. A PBSI template (Concure Oncology, Seattle, WA, USA) is attached to the fiducial needle and immobilized using a modified medical articulated arm (Fisso, Medtec Baitella Alt, Switzerland). The preloaded needles containing ^103^Pd strands are then inserted under US guidance [33]. In patients allocated to receive spacer, an amount of 4–10 cc of biodegradable hyaluronic acid (Barrigel™, Palette Life Sciences, Santa Barbara, CA, USA or Restylane SubQ®, Galderma Benelux, Breda, the Netherlands) is injected directly under the skin under ultrasound guidance covering the PTV projection, aiming to create an extra 0.5–1 cm space between the treatment volume and the skin. If the skin is judged not to be at risk in all projection quadrants, it could be decided to only inject the area at risk. The injected skin quadrants will be reported specifically. All radiation oncologists involved in this study are trained to perform the intervention and the injection procedure and reporting instructions are incorporated in trial protocols. The hyaluronic acid spacer is expected to be fully degraded after 3–9 months.

### Outcomes

The primary endpoint of this trial is the occurrence of telangiectasia at two years after PBSI. Assessment is performed by a blinded physician, following the Bentzen’s 4-point scale which is included in the LENT/SOMA questionnaire [34, 35]. This scale is defined as: “none;” grade I, “< 1 cm^2^,” grade II, “1–4 cm^2^;” and grade III, “> 4 cm^2^.” Patients will also be blinded for the allocated treatment. The secondary outcomes include the local recurrence rate at five and ten years, the disease-free and overall survival rates at five years, as well as brachytherapy and spacer injection adverse events (AE) according to the commonly used NCI Common Toxicity Criteria for Adverse Events (CTCAE v 4.03) scale for acute side effects [36], practically occurring within three months of the brachytherapy, and the Radiation Therapy Oncology Group (RTOG)/ European Organisation for Research and Treatment of Cancer (EORTC) scoring systems for late side effects [37], practically occurring after three months. The symptoms include the experience of pain, skin redness, pigmentation, induration, dermatitis, subcutaneous induration, and the occurrence of infection at the site of spacer injection. Additionally, patient-reported outcome measures (PROMs) include the cosmetic result with the Breast Cancer Treatment Outcome Scale (BCTOS) questionnaire [38], using a validated Dutch version which will shortly be published by our group [39] and the health-related Quality of Life using the Dutch version EORTC QLQ-C30 and BR23 questionnaires, version 3 [40]. Ipsilateral breast recurrence must be proven getting a copy of the biopsy or the salvage surgery pathology report. Dosimetry outcomes include the PTV volumes receiving at least 100% or 200% of the prescribed dose (V_100_ and V_200_) as quality assurance for all treatment plans and maximum dose to a small skin volume of 2 mm thickness over 1 cm^2^ (D_0.2cc_) [21, 41] and the presence of a hotspot (isodose ≥ 90%) [42] on 1 cm^2^ of the skin as indicators of skin toxicity risk.

**Fig. 1:**
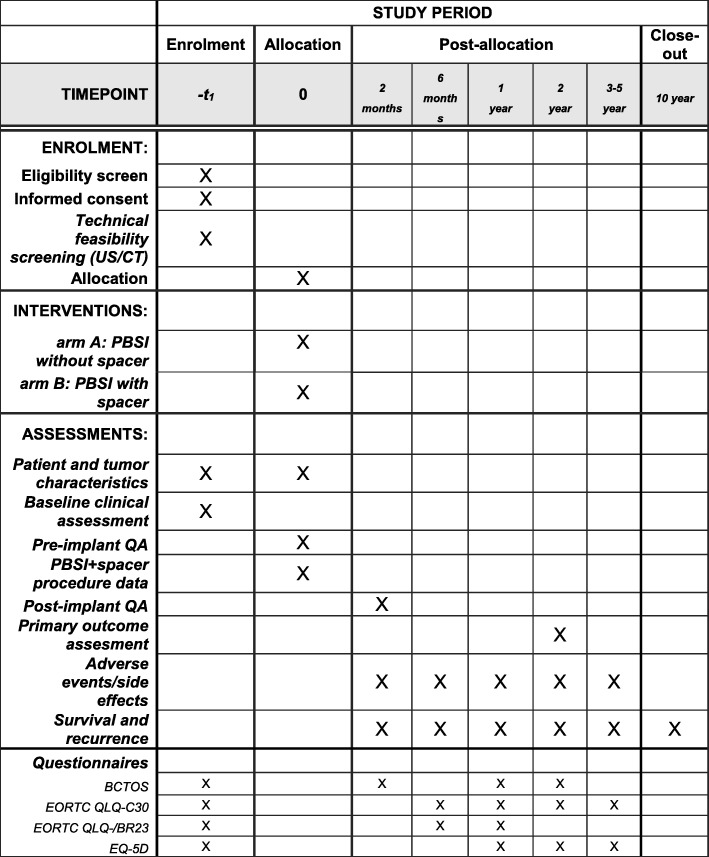
Schedule of enrolment, interventions, and assessments in this study

Outcomes are collected before the PBSI implantation at baseline, at the end of the procedure, and at two months, six months, and every year up to five years, during follow-up visits at the cancer center. If a patient does not attend a follow-up appointment, she will be called and/or her family doctor contacted. Reason for no-show will be recorded in order to ensure exhaustive capture of survival, recurrence, and/or AEs. Overall and disease-specific survival will be assessed until 10 years through GBA (Population registry, Gemeentelijke Basis Administratie) and/or general practitioners. A summary of the timing of questionnaires is detailed in Fig. 1.

### Sample size

Previous studies found 22.4% telangiectasia at two years [9]. We expect the use of a spacer could reduce the skin dose to 50% [21] and the occurrence of any telangiectasia (≥ grade 1) to 7.7% [43]. To test this reduction (e.g. the superiority of the intervention), 105 (Fisher’s exact test) patients per treatment arm would be needed (α = 0.05, β = 0.20). A sample size of 230 patients will allow for a 10% lost to follow-up rate.

### Recruitment

The Erasmus MC - Cancer Institute treats approximately 1000 patients with adjuvant breast radiotherapy every year. Given the inclusion criteria, it is expected that approximately 20% of these patients are eligible for PBSI. On top of this, referrals from outside the area are also expected specifically for PBSI. This makes it very likely that the required sample size could be recruited in three years.

### Treatment allocation and blinding

After written informed consent and final eligibility check, the radiation oncologist will enroll the patient and randomization will be performed by the department’s independent trial manager. Patients will be randomly allocated to one of the treatment arms (spacer injection or no spacer injection) in a 1:1 allocation ratio, applying a variable block size randomization (block sizes 2, 4, and 6). This concealed allocation will be computer-generated using the online randomization tool ALEA.

Patients will be blinded for the allocated treatment, as the spacer injection is performed under sedation. However, in some cases the patient might see or feel the effect of the spacer injection later. The treating radiation oncologist will be blinded during treatment planning and during the implant of the palladium seeds and be unblinded after the implant to inject the spacer or not using a telephone call with the departments trial manager.

Investigators will be blinded for allocated treatment during assessment of primary endpoint by performing this assessment in a separate visit in which the physician does not have access to the patient’s medical file. Unblinding will be performed if a patient is going (un)planned off-study. In addition, in case of medical emergencies possibly caused by the spacer, unblinding will be performed. In these cases, a patient’s allocated treatment can be unblinded by checking the medical record of the implantation or by contacting the trial management.

### Data management

Secure collection of data is performed. Data entry will be performed using a predefined case report form (CRF) (Additional file 2) with accompanied data entry protocol. This provides instructions units to be used, missing data handling and range checks.

### Statistical methods

All statistical tests will be two-sided and a *p* value < 0.05 is considered to be significant. Statistical analyses will be performed using IBM SPSS version 24 (IBM Corporation, Armonk, NY, USA). Data will be analyzed following intention-to-treat and per-protocol. Missing data will be handled using multiple imputation. Descriptive statistics will be used for all outcome measures.

A Fisher’s exact test or Chi-squared test will be performed to test the reduction in the rate of telangiectasia in the study groups at two-year follow-up, i.e. to test the hypothesis that the rates of telangiectasia in both study groups are equal (superiority study).

Local recurrence-free survival as well as overall and disease-specific survival rates at five and ten years will be estimated using the Kaplan–Meier method. The local recurrence rate will be reported at five and ten years. A Fisher’s exact test or Chi-squared test will be performed to test the difference in proportions (six-month, one-year and two-year cumulative rate of side effects, skin dose > 90% over at least 1 cm^2^ at post-planning) between groups. (Skin) dosimetry data will be compared using a Mann–Whitney U test or an unpaired Student’s t-test depending of distribution of data. To study the effect of spacer on cosmesis (BCTOS questionnaire) and quality of life (EORTC-QLQ-C30/BR23 and EQ5-D questionnaire) over time, a repeated measurements analysis will be performed (linear mixed model, covariance structure: unstructured) with independent variables time, allocated group, and interaction effects between time and allocated group.

## Discussion

For early stage breast cancer patients that have outstanding survival outcomes [3, 4], the role of radiotherapy is essentially cosmetic [5]. The skin is a critical structure in breast radiotherapy, with skin dose as the main risk factor [21, 22]. In this study, we test an intervention to reduce cosmetic impairment by aiming to prevent long-term skin toxicity.

Telangiectasia are a specific marker of radiation toxicity [21, 22]. Although rates are lower than with whole breast irradiation, in breast brachytherapy 10–27% [9, 19, 24] of the patients develop some grade of telangiectasia. Rates of telangiectasia normally peak at two years till it stabilizes. Most of the lesions are permanent resulting in decreased quality of life [9]. Other skin toxicity scales (pigmentation, induration, fibrosis) are less specific for capturing radiation induced side effects [21].

Among our secondary outcomes are standard oncological outcomes. Based on our pre-clinical study we do not expect the spacer to influence the oncological effectivity of PBSI. [44]. This work in mastectomy specimens showed excellent feasibility of creating an extra 5-mm space directly below the skin using a biodegradable spacer. This space is not part of the PTV in LDR seed brachytherapy as the CTV expansion is limited to 5 mm below the skin by protocol [33]. The spacer partly lifted the skin, but also moved the breast tissue inferior and laterally. However, with the seeds already in place, we expect that any change in PTV geometry will be similar in the treated volume containing the seeds. This hypothesis was supported by the excellent and comparable PTV coverage (V_100%_) before and after injection in the pre-clinical study [44]. However, this finding should be confirmed in a clinical setting.

Other secondary outcomes are brachytherapy and spacer injection AEs according to commonly used NCI CTCAE and RTOG/EORTC scoring systems for late side effects. Where our main hypothesis is that the spacer increases distance and reduces skin dose and telangiectasia, this will allow for analyzing the effect on other less radiotherapy-specific symptoms such as pain, skin redness, pigmentation, induration, dermatitis, subcutaneous induration, and the occurrence of infection at the site of spacer injection. Although hyaluronic acid is widely used as a dermal filler, the application as a skin spacer in patients treated with breast radiotherapy is a new concept and any unexpected side effects will be captured. Skin dose outcomes will potentially lead to updated skin dose constraints in treatment planning. Also, it could distinguish radiotherapy induced toxicity from other causes (i.e. intervention-related toxicity). PROMs assess the effect of the skin spacer on cosmesis, function, and quality of life. Furthermore, by using internationally recognized PROMs, a better comparison with other radiotherapy techniques is possible.

This clinical trial was designed because it is unknown whether the dosimetric benefit of the spacer, which was found in our pre-clinical study [44], translates in a real patient benefit. An example of a clinical trial that could not demonstrate that a dosimetric benefit translates into better patient outcomes, is the breast intensity modulated radiation therapy (IMRT) trial. In this trial, the improved radiation dose distribution and reduced moist desquamation using IMRT, compared to standard wedge RT, did not result in reduced long-term side effects such as chronic breast pain [43].

Our primary analysis will be done following the intention-to-treat principle: the effect of skin spacer on telangiectasia rate at two years. However, a per-protocol analysis will allow for a better definition of a successful skin spacer as the skin spacer injection protocol (> 5 mm in a PTV skin projection area) is not definite and the trial could be hypothesis-generating.

A drawback of our study is that we are not able to secure a full double-blind design. Patients might be aware of an injected spacer as it could be palpable under the skin. For physicians, it might be possible to remember the allocated treatment after being unblinded during the PBSI procedure. However, with the assessment of the primary outcome at two-year follow-up, this memory effect is not likely to cause any bias. Also, the type of outcome measure (telangiectasia using Bentzen’s 4-point scale) allows for an objective, reproducible assessment. Furthermore, this a single-center study and our findings should be confirmed in a multicenter setting. Lastly, with only patients undergoing PBSI in this study, generalization of our findings to other APBI techniques should be done with caution. However, theoretically, this principle would hold for any APBI technique with a rapid dose fall off.

In this trial, we investigate the effect of a subcutaneous spacer injection on the skin dose, late skin toxicity, and cosmetic outcome in patients treated with PBSI in the setting of breast-conserving therapy. Our results will be relevant for most forms of breast brachytherapy as well as robotic radiosurgery, as skin spacers could be used to protect the skin with these other techniques.

## Trial status

Protocol version 5, 26 March 2018. The first patient was enrolled in the study on 8 September 2017. Expected completion of recruitment is at the end of 2020.

## Additional files

Additional file 1: SPIRIT 2013 checklist. Recommended items to address in a clinical trial protocol and related documents. (PDF 117 kb)

Additional file 2: CRF template. A predefined case report form provides instructions units to be used, missing data handling and range checks. (PDF 415 kb)

## Abbreviations

APBI: Accelerated partial breast irradiation
BCTOS: Breast Cancer Treatment Outcome Scale
CDC: Centers for Disease Control and Prevention
CRF: Case report form
CT: Computed tomography
CTCAE: Common Toxicity Criteria for Adverse Events
CTV: Clinical target volume
D_0.2cc_: Maximum dose to a small skin volume of 0.2cc
DCIS: Ductal carcinoma in situ
EORTC: European Organisation for Research and Treatment of Cancer
GEC-ESTRO: Groupe Européen de Curiethérapie and European SocieTy for Radiotherapy & Oncology
HA: Hyaluronic acid
IDC: Invasive ductal carcinoma
IMRT: Intensity modulated radiation therapy
LDR: Low dose rate
LENT/SOMA: Late effects of normal tissues, subjective, objective, management, analytical
MREC: Medical research ethics committee
NSAID: Non-steroidal anti-inflammatory drugs
PBSI: Permanent breast seed implant
PROM: Patient-reported outcome measure
PTV: Planned target volume
RTOG: Radiation Therapy Oncology Group
SPIRIT: Standard Protocol Items: Recommendations for Interventional Trials
US: Ultrasound
V_100_: Volume of PTV receiving at least 100% of prescribed dose
V_200_: Volume of PTV receiving at least 200% of prescribed dose
