# Supplementary material for: A randomized controlled trial testing a hyaluronic acid spacer injection for skin toxicity reduction of brachytherapy accelerated partial breast irradiation (APBI): a study protocol
Source: Trials. 2018 Dec 17;19:689. doi: 10.1186/s13063-018-3035-3 (PMC6298013; doi:10.1186/s13063-018-3035-3)
Supplement: Supplementary file 2 — CRF template. A predefined case report form provides instructions units to be used, missing data handling and range checks. (PDF 415 kb) [file 13063_2018_3035_MOESM2_ESM.pdf]

EMC 16-400

A MULTICENTER RANDOMIZED CONTROLLED TRIAL TESTING HYALURONIC ACID SPACER INJECTION FOR SKIN TOXICITY REDUCTION OF PERMANENT BREAST SEED IMPLANT (PBSI)

CASE REPORT FORM (CRF)

A MULTICENTER RANDOMIZED CONTROLLED TRIAL TESTING HYALURONIC ACID SPACER INJECTION FOR SKIN TOXICITY REDUCTION OF PERMANENT BREAST SEED IMPLANT (PBSI)

Principal Investigator: Prof. dr. J.P. Pignol

Co-investigator: Drs. G. Struik

Protocol version: Mar 2018 (version 5)

Note: this CRF was built as an e-CRF in OpenClinica

Overview of events and CRF

The CRF's are devided over the following events:

- Baseline
- Treatment
- Follow-up 2M, 6M, 1YR, 2YR, 3YR, 4YR, 5YR, 10YR
- Recurrence
- Off-study

Randomization Form will be built separately in ALEA (*)

This form will contain basic patient characteristics, and a selection of the eligibility criteria:

- Date of birth➔ calculated age, ≥ 50 years (**yes**/no)
- Surgery: BCS+ axillary lymph node dissection or BCS + sentinel lymph node biopsy, (**yes**/no)
- *Histological* diagnosis: IDC or DCIS, (**yes**/no)
- *Conditional question:*
    - if IDC: Surgical margins clear at ink or re-excision negative (**yes**/no)
    - if DCIS: Surgical margin ≥ 2mm or re-excision negative (**yes**/no)
- Tumor size (mm), ≤30mm (**yes**/no)
- Nodal status (positive/**negative**)
- Lymphovascular invasion (yes/**no**)
- Known allergy for hyaluronic acid (yes/**no**)
- Neo-adjuvant chemotherapy (yes/**no**)
- PBSI technically feasible (**yes**/no)
- Any other exclusion criterion (yes/**no**)
- Date of written informed consent

- Date of randomization
- Randomization result
- Subject IDnr

(*) export of data any time in agreement with CTC

PROMs will be collected in ABC Zorgmonitor: QLQ-C30/BR23, EQ-5D, BCTOS

Additional questionnaires not yet in Zorgmonitor: **BCTOS at BL, 2M, YR1 and YR2**

A MULTICENTER RANDOMIZED CONTROLLED TRIAL TESTING HYALURONIC ACID SPACER INJECTION FOR SKIN TOXICITY REDUCTION OF PERMANENT BREAST SEED IMPLANT (PBSI)

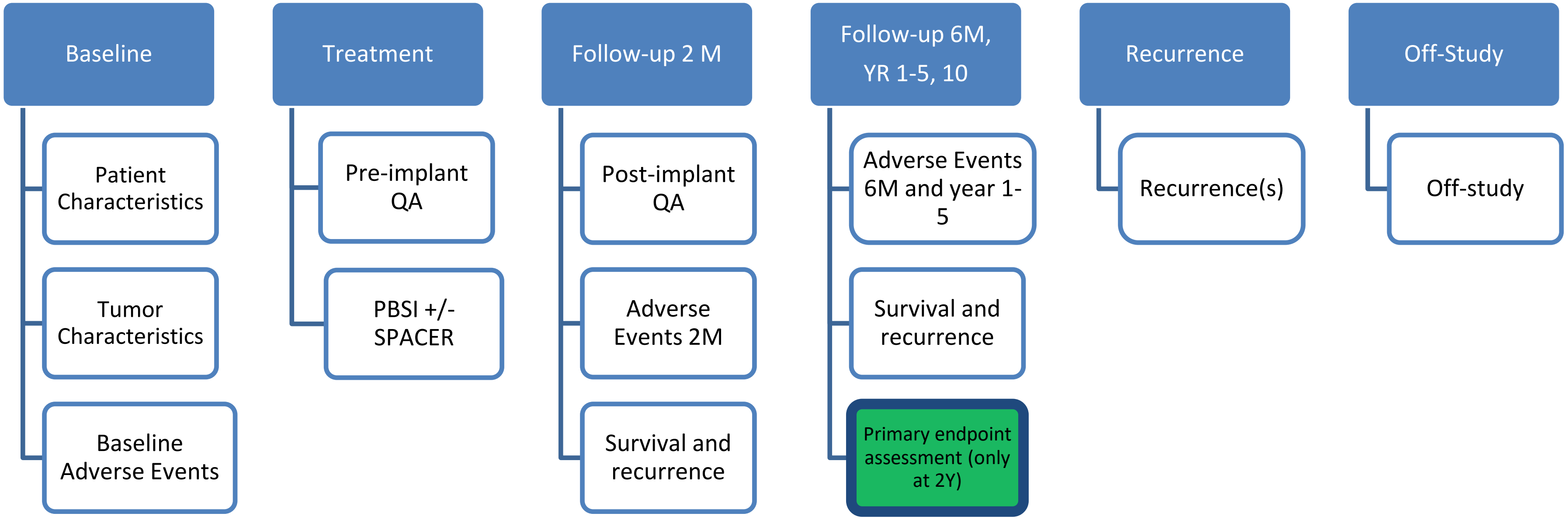

EMC 16-400

A MULTICENTER RANDOMIZED CONTROLLED TRIAL TESTING HYALURONIC ACID SPACER INJECTION FOR SKIN TOXICITY REDUCTION OF PERMANENT BREAST SEED IMPLANT (PBSI)

CRF: Patient Characteristics

[DOR] Date of randomization DD / MM/ YYYY

[AGE] Age at registration |__|__|__|

[ALLINCL] Did patient meet all inclusion criteria? |__| *0 =no; 1 = yes*

[ALLEXCL] Did patient meet any exclusion criteria? |__| *0 =no, 1 = yes*

If ALLINCL=1 or ALLEXCL=1:
[ELIGCOM] Comments on eligibility [FREE TEXT]

[DDIAG] Date of first histological diagnosis DD/ MM/ YYYY

[HEIGHT] Height |__|__|__| cm

[WEIGHT] Weight |__|__|__| kg

[CUPSIZE] Breast cup size |__ __ __|__| (f.e. 80B)

[SMOKSTAT] Smoking status |__|
0 = never smoked
1 = former smoker
2 = current smoker
9 = unknown

[DM] Diabetes Mellitus |__|
0 = no
1 = insulin dependent
2 = not insulin dependent

[HT] Hypertension |__| *0=no; 1=yes*

[CVRF] History of MI, CVA, PAOD? |__| *0=no; 1=yes*

[ADJTX] adjuvant therapy before/after PBSI |__|
0 = no
1 = chemo
2 = hormone
3 = chemo+ hormone

If ADJTX>0:

[STARTHTX]	Start date hormone therapy	DD / MM / YYYY
[STARTCTX]	Start date chemotherapy	DD / MM / YYYY
[STOPCTX]	Date of last chemotherapy	DD/ MM/ YYYY

[SURGDATE]	Date of surgery:		DD / MM / YYYY
[SURGTYPE]	Type of surgery:	|__|	1 = BCS + axillary lymphnode dissection<br>2 = BCS + sentinel lymphnode biopsy
[SSI]	Surgical Site Infection (CDC)	|__|	0 = none<br>1 = superficial incisional SSI<br>2 = deep incisional SSI

CRF: Tumor Characteristics

[T]	T (NM)-classification 1997	T|__|	
[N]	(T)N(M)-classification 1997	N|__|	
[M]	(TN)M-classification 1997	M|__|	
[STAGE]	Clinical Stage	|__|	0 = stage 0<br>1 = stage 1<br>11 = stage 1b<br>2 = stage 2a<br>22 = stage 2b<br>3 = stage 3a<br>33 = stage 3b<br>333 = stage 3c<br>4 = stage 4

[TUMLAT]	Tumor laterality	|__|	1 = *left*<br>*2 = right*
[TUMSITE]	Tumor anatomical subsite	|__|	*1= nipple*<br>*2 = central*<br>*3 = upper- inner quadrant*<br>*4 = lower- inner quadrant*<br>*5 = upper-outer quadrant*<br>*6 = lower-outer quadrant*<br>*7= axillary tail*
[TUMORSIZE]	Tumor size	|__|__| mm	
[DISTSKIN]	Distance tumor to skin	|__|__|__| mm	
[MARGIN]	Resection margin status	|__|	*0 = tumor free at ink (IDC) or >2mm for DCIS*<br>*1 = focal irradical or <2mm for DCIS*<br>*2 =irradical*<br>*9 = unknown*
[HISTOL]	Histological Diagnosis	|__|	*1 = IDC*<br>*2 = DCIS*<br>*3 = IDC+DCIS*<br>*4 = other, ……*
[HISTCOM]	*IF HISTOL=4 please specify FREE TEXT*		
[TUMGRAD]	Modified Scarf-Bloom-Richardson grade	|__|	
[PR]	PR-receptor status	|__|	*0 = negative*<br>*1 = positive*
[ER]	ER-receptor status	|__|	*0 = negative*<br>*1 = positive*
[HER2NEU]	Her-2neu -receptor status	|__|	*0 = negative*<br>*1 = positive*
[LVI]	Lymfovascular Invasion	|__|	*0 = no*<br>*1 = yes*

[MULTI]	multicentricity	|__|	*0 = no*<br>*1 = yes*
[EXTDCIS]	Extensive DCIS (beyond invasive tumor, or >3cm)	|__|	*0 = no*<br>*1 = yes*
[BILAT]	*Bilateral breast cancer?*	|__|	*0 = no*<br>*1 = yes*
[RECUR}	*Recurrent breast cancer?*	|__|	*0 = no*<br>*1 = yes*
[ACTOTH]	*Active other cancer (defined by malignancy in<5 year?)*	|__|	*0 = no*<br>*1 = yes*

CRF: Baseline Adverse Events (as close as possible to start PBSI)

[AEDATE] Date of AE assessment DD / MM/ YYYY

[BASPAIN] Baseline pain in breast |__| 0 = none
1 = occasional and minimal, hypersensation, pruritus
2 = intermittent and tolerable
3 = persistent and intense
4 = refractory and excruciating

[BASSSI] Baseline Surgical Site Infection |__| 0 = none
1 = superficial SSI
2 = deep SSI

[BASTELEANG] Baseline Teleangiectasia (Bentzen scale) |__| *0 =none*
1 = grade I – less than 1cm2
2 =grade II – 1 to 4 cm2
3 =grade III – over 4 cm2

[BASSUBINDUR]Baseline Subcutaneaous Induration |__| *0 =none*
1 = slight induration and loss of subcutaneous fat
2 =moderate fibrosis but asymptomatic
3 =severe induration and loss of subcutaneous tissue; field contracture
4 =necrosis

[BASPIGM] Baseline pigmentation |__| 0 = none
1 = transitory, slight
2 = permanent, marked

[OTHERAE] Any other AE? |__| 0=no, 1=yes *If yes, enter in grid/table (multiple entries possible):

- System organ Class (conform CTCAE4/MEDRA)
- AE term (conform CTCAE4/MEDRA)
- CTC grade (1-4)

EMC 16-400

A MULTICENTER RANDOMIZED CONTROLLED TRIAL TESTING HYALURONIC ACID SPACER INJECTION FOR SKIN TOXICITY REDUCTION OF PERMANENT BREAST SEED IMPLANT (PBSI)

CRF: Pre-implant QA

Group: General

[PLANCT1] Date of planning CT scan DD / MM / YYYY

[PRECTV] CTV |__|__|.|__| cc

[PREPTV] PTV |__|__|__|.|__| cc
[PRESEEDNUM] Total number of seeds |__|__||__|

[PRENEEDNUM]Total number of needles |__|__||__|

[TOTACT] Total activity of seeds |__|__||__|.|__|U

[PRESKINPTV] minimal distance skin to PTV |__|__|.|__|mm

[PRESKINISO] distance between CTV isocenter and skin |__|__|.|__|mm

Group: DVH parameters

[PREV100] Pre-implant V100 |__|__|__|.|__| %
[PREV200] Pre-implant V200 |__|__|__|.|__| %

[PRED90] Pre-implant D90 |__|__|__|.|__| cc

[PRESK90] Pre-implant skin isodose>90% over 1cm^2 |__| 0=no, 1=yes

[PRED0.2] Pre-implant D0.2cc skin dose |__|__|__|.|__| Gy
[PRED0.05] Pre-implant D0.05cc skin dose |__|__|__|.|__| Gy

CRF: PBSI +/- SPACER

[PBSIDATE] Date of PBSI procedure DD / MM / YYYY

[PBSIDUR] Duration of PBSI procedure |__|__|__|min

[PBSIDIFF] any difficulties in PBSI procedure, please specify……………………………………………

[SPACER] Spacer administered |__| *0=no; 1=yes*

[SPACDUR] Duration of spacer injection procedure |__|__|__|min

[SPACMCR] Spacer administered in mediocranial quadrant of skin projection |__| *0=no, 1=yes*

[SPACMCA] Spacer administered in mediocaudal quadrant of skin projection |__| *0=no, 1=yes*

[SPACLCA] Spacer administered in laterocaudal quadrant of skin projection |__| *0=no, 1=yes*

[SPACLCR] Spacer administered in laterocranial quadrant of skin projection |__| *0=no, 1=yes*

If SPACER=1: [SPACVOL] Volume of spacer administered |__|__|__|__| cc

(enter 9999 if not recorded)

[SPACDIFF] any difficulties in spacer injection, please specify:……………………………………………………

[USSPATRANCEN] mid spacer thickness central transversal plane on ultrasound |__|__|.|__| mm

[USSPATRANCRA] mid spacer thickness cranial transversal plane on US |__|__|.|__|mm

[USSPATRANCAU] mid spacer thickness caudal transversal plane on US |__|__|.|__|mm

[USSPASAGCEN] mid spacer thickness central saggital plane on US |__|__|.|__|mm

[USSPASAGLAT] mid spacer thickness lateral saggital plane on US |__|__|.|__|mm

[USSPASAGMED] mid spacer thickness medial saggital planel on US |__|__|.|__|mm

[SPASUC] Spacer injection successful (>5mm in all injected quadrants) |__| *0=no, 1=yes*

CRF: treatment pain

[PAINBAS]	Pain before procedure (baseline, LENTSOMA)	|__|	*0-4*
[PAINPBSI]	Pain during procedure (baseline, LENTSOMA)	|__|	*0-4*
[PAINPOST]	Maximum pain after procedure (1-2 d post-implant, LENTSOMA)	|__|	*0-4*

CRF: Adverse Events 2 months

[2MASSDATE]	Date of AE assessment	DD / MM / YYYY	
[2MPAIN]	pain in breast	|__|	0 = none<br>1 = occasional and minimal, hypersensation, pruritus<br>2 = intermittent and tolerable<br>3 = persistent and intense<br>4 = refractory and excruciating
[2MPAINS]	If AEPAIN>0: Was AE serious(SAE)?	|__| 0=no, 1=yes	
[2MRED]	Redness	|__|	0 =none<br>1 = yes but no effect on ADL<br>2 = yes and effect on ADL
[2MREDS]	If AERED >0: Was AE serious(SAE)?	|__| 0=no, 1=yes	
[2MPIGM]	Pigmentation	|__|	0 = none<br>1 = transitory, slight<br>2 = permanent, marked
[2MPIGMS]	If AEPIGM >0: Was AE serious(SAE)?	|__| 0=no, 1=yes	
[2MSKININD]	Skin induration	|__|	0 = none<br>1 = Mild induration, able to move skin parallel to plane (sliding) and perpendicular to skin (pinching up)<br>2 = Moderate induration, able to slide skin, unable to pinch skin; limiting instrumental ADL<br>3 = Severe induration, unable to slide or pinch skin; limiting self-care ADL<br>4 = Generalized
[2MSKININDS]	If AESKININD >0: Was AE serious(SAE)?	|__| 0=no, 1=yes	

[2MRADDERM] Radiation dermatitis |__| 0 = none
1 = Faint erythema or dry desquamation
2 = Moderate to brisk erythema; patchy moist desquamation, mostly confined to skin folds and creases; moderate edema
3 = Moist desquamation in areas other than skin folds and creases; bleeding induced by minor trauma or abrasion
4 = skin necrosis/ulceration of full thickness dermis; spontaneous bleeding from involved site; (skin graft indicated)

[2MRADDERMS] If AERADDERM >0:
Was AE serious(SAE)? |__| 0=no, 1=yes

[2MSKINTOXDIST] Distance of clinical max skintoxicity from reference point (nipple lowerinner) |__|__|__|mm
[2MSKINTOXANGLE] Angle of clinical max skintoxicity from reference point (nipple lowerinner) |__|__|__| 0-360°

[2MSSI] Surgical Site Infection |__| 0 = none
1 = superficial SSI
2 = deep SSI

[2MSSIS] If AESSI >0: Was AE serious(SAE)? |__| 0=no, 1=yes

[2MOTHERAE] Any other RT (or spacer) induced AE? |__| *0=no; 1=yes*

If OTHERAE=yes: Enter in grid/table (multiple entries possible):

- System organ Class (drop down list conform CTCAE4/MEDRA)
- AE term (drop down list conform CTCAE4/MEDRA)
- Worst grade observed since last AE evaluation (1-5)
- Serious: yes/no

CRF: Post-implant QA

Group: general postplanning CT variables

[PLANCT2] Date of post-implant CT scan DD/ MM/ YYYY

[POSTCTV] post-implant CTV |__|__|.|__| cc

[POSTVOL] post-implant treatment volume with Minimal peripheral dose (90Gy) |__|__|__|.|__| cc

[POSTSEEDNUM] Total number of seeds |__|__||__|

[SEEDDISPLACED] any seeds displaced>1 cm |__| 0=no, 1=yes

[DISPSPEC] *If* SEEDDISPLACED *=1:* Specify [FREE TEXT]

[POSTVOLSKIN] minimal distance skin to POSTVOL |__|__|.|__|mm

[POSTISOSKIN] distance between CTV isocenter and skin |__|__|.|__|mm

Group: DVH parameters on CT

[POSTV100] post-implant V100 |__|__|__|.|__| %

[POSTV200] post -implant V200 |__|__|__|.|__| %

[POSTD90VOL] post-implant D90 of skin |__|__|__|.|__| cc

[POSTSK90] post-implant skin isodose>90% over 1cm^2 |__| 0=no, 1=yes

[POSTD02] post-implant D0.2cc skin dose |__|__|__|.|__| Gy
[POSTD005] post-implant D0.05cc skin dose |__|__|__|.|__| Gy

Group: spacer measurements on CT at 2 months

[CTSPACER] any spacer visible on CT |__| *0=no, 1=yes*

[CTSPACMCR] spacer in mediocranial quadrant of skin projection |__| *0=no, 1=yes*

[CTSPACMCA] spacer in mediocaudal quadrant of skin projection |__| *0=no, 1=yes*

[CTSPACLCA] spacer in laterocaudal quadrant of skin projection |__| *0=no, 1=yes*

[CTSPACLCR] spacer in laterocranial quadrant of skin projection |__| *0=no, 1=yes*

If SPACER=1:

[CTSPACVOL] Volume of spacer on CT |__|__|__|__| cc

[CTSPATRANCEN] mid spacer thickness central transversal plane on CT |__|__|.|__| mm

[CTSPATRANCRA] mid spacer thickness cranial transversal plane on CT |__|__|.|__|mm

[CTSPATRANCAU] mid spacer thickness caudal transversal plane on CT |__|__|.|__|mm

[CTSPASAGCEN] mid spacer thickness central saggital plane on CT |__|__|.|__|mm

[CTSPASAGLAT] mid spacer thickness lateral saggital plane on CT |__|__|.|__|mm

[CTSPASAGMED] mid spacer thickness medial saggital plane on CT |__|__|.|__|mm

Group: gafchromic film skin dose measurement

[FILMUSED] gafchromic film used? |__| 0=no, 1=yes

IF FILMUSED=1 than

[FILMMAX]	maximum point skindose on film	|__|__|__|.|__| Gy
[FILMMAX1CM]	maximum skindose on film over at least 1cm^2	|__|__|__|.|__| Gy
[FILMAUC]	AUC skindose on film	|__|__|__|.|__| Gy*cm
[FILMLOCDIST]	Distance of max point skindose on film from reference point (projected isocenter)	|__|__|__|.|__| mm
[FILMLOCANG]	Angle of max point skindose on film from reference point (projected isocenter)	|__|__|__| 0-360°

CRF: Survival and recurrence

[FUDATE]	Date last known to be alive / date of death	DD/MM/ YYYY
[SURVIVAL]	Survival status	|__| *1 = alive*<br>*2 = death*<br>*8 = lost to follow-up*
[RADDATE]	Date last mammography or CT-scan	DD/MM/YYYY [ ]not done
[RECURR]	Any recurrent disease (not yet reported)<br>*If yes, please complete the recurrence form*	|__| *0=no; 1=yes*

CRF: Recurrence

Please report/update all recurrences until start of a new anticancer treatment

[LREC] **Local recurrence *(ipsilateral breast/chestwall)*** |__| *0=no; 1=yes*

If LREC=1:

[LRECDATE] Date of local recurrence DD/MM/ YYYY

[LRECLOC] Localization of local recurrence |__|
1 = ipsilateral breast same quadrant
2 = ipsilateral breast other quadrant
3 = ipsilateral chest wall same quadrant
4 = ipsilateral chest wall other quadrant
5 = ipsilateral, location not documented

[LRECHIS] Cyt/hist proof of recurrence |__| *0 = no; 1=yes*

[LRECPATH] Local recurrence of disease under study confirmed by pathologist |__|
0 = no, (probably) a new primary tumor
1 = yes, (probably) a local recurrence
2 = uncertain, specify

[SLRECDIA] Comments on diagnosis [FREE TEXT]

[RREC] **Regional recurrence *(ipsilateral lymphnodes/axilla)*** |__| *0=no; 1=yes*

If RREC=1:

[RRECDATE] Date of regional recurrence DD/MM/ YYYY

[RRECLOC] Localization of regional recurrence |__|
1 = internal mammary chain
2 = supraclavicular area
3 = axilla

[RRECHIS] Cyt/hist proof of regional recurrence |__| *0 = no; 1=yes*

[RRECPATH] Regional recurrence of disease under study confirmed by pathologist |__| *0 = no, (probably) other tumor*

				1 = yes, (probably) regional recurrence<br>*2 = uncertain, specify*
	[SRRECDIA]		[FREE TEXT]	
[DMET]	**Distant metastases**		|__|	*0=no; 1=yes*
	If DMET=1:			
	[DMETDATE]	Date of distant metastases	DD/MM/ YYYY	
	[DMETHIS]	Cyt/hist proof of distant metastases	|__|	*0 = no; 1=yes*
	[DMETPATH]	Distant metastases from disease under study confirmed by pathologist	|__|	*0 = no, (probably) (from) other primary tumor*<br>*1 = yes, (probably)from disease under study*<br>*2 = uncertain, specify*
	[DMETDIA]	Comments on diagnosis	[FREE TEXT]	
[PLANTRTM]	Planned further treatment<br><br>*(only first anticancer treatment after PBSI)*		|__|	*0= none*<br>*1= mastectomy*<br>*2= radiotherapy*<br>*3= chemotherapy*<br>*4= targeted therapy*<br>*5= combination*
[SPLANTRTM]	Specify: ___________________________		[FREE TEXT]	

If locoregional or distant recurrence, follow-up local recurrence

EMC 16-400

A MULTICENTER RANDOMIZED CONTROLLED TRIAL TESTING HYALURONIC ACID SPACER INJECTION FOR SKIN TOXICITY REDUCTION OF PERMANENT BREAST SEED IMPLANT (PBSI)

CRF: Adverse Events 6 months and year 1-5

[AEDATE]	Date of AE assessment	DD / MM / YYYY	
[AETELEANG]	Telangiectasia (Bentzen scale)	|__|	*0 =none*<br>*1 = grade I – less than 1cm2*<br>*2 =grade II – 1 to 4 cm2*<br>*3 =grade III – over 4 cm2*
[AETELEANGS]	If AETELEANG >0: Was AE serious(SAE)?	|__|	0=no, 1=yes
[AEPAIN]	pain in breast	|__|	0 = none<br>1 = occasional and minimal, hypersensation, pruritus<br>2 = intermittent and tolerable<br>3 = persistent and intense<br>4 = refractory and excruciating
[AEPAINS]	If AEPAIN>0: Was AE serious(SAE)?	|__|	0=no, 1=yes
[AERED]	Redness	|__|	0 =none<br>1 = yes but no effect on ADL<br>2 = yes and effect on ADL
[AEREDS]	If AERED >0: Was AE serious(SAE)?	|__|	0=no, 1=yes
[AEPIGM]	Pigmentation	|__|	0 = none<br>1 = transitory, slight<br>2 = permanent, marked
[AEPIGMS]	If AEPIGM >0: Was AE serious(SAE)?	|__|	0=no, 1=yes
[AESKININD]	Skin induration	|__|	0 = none<br>1 = Mild induration, able to move skin parallel to plane (sliding) and perpendicular to skin (pinching up)

			2 = Moderate induration, able to slide skin, unable to pinch skin; limiting instrumental ADL<br>3 = Severe induration, unable to slide or pinch skin; limiting self-care ADL<br>4 = Generalized
[AESKININDS]	If AESKININD >0: Was AE serious(SAE)?	|__|	0=no, 1=yes
[AESUBIND]	Subcutaneaous Induration	|__|	*0 =none*<br>*1 = slight induration and loss of subcutaneous fat*<br>*2 =moderate fibrosis but asymptomatic*<br>*3 =severe induration and loss of subcutaneous tissue; field contracture*<br>*4 =necrosis*
[AESUBINDS]	If AESUBIND >0: Was AE serious(SAE)?	|__|	0=no, 1=yes
[OTHERAE]	Any other RT (or spacer) induced AE?	|__|	*0=no; 1=yes*

If OTHERAE=yes: Enter in grid/table (multiple entries possible):

- System organ Class (drop down list conform CTCAE4/MEDRA)
- AE term (drop down list conform CTCAE4/MEDRA)
- Worst grade observed since last AE evaluation (1-5)
- Serious: yes/no

CRF: Off-study

[DOFF]	Date off-study		DD / MM / YYYY
[OFFREAS]	Reason off-study	|__|	*1 = end of follow-up period, without recurrence*<br>*2 = recurrent disease*<br>*3 = start systemic/locoregional anti-cancer treatment/ breast surgery*<br>*4 = death*<br>*5 = patient withdrawal*<br>*6 = ineligibility*<br>*7 = other*
[SPOFFREAS]	Specify reason off-study		[FREE TEXT]

If OFFREAS=3:

[CODEATH]	Cause of death	|__|	*1 = Breast cancer*<br>*2 = Concurrent disease*<br>*3 = Treatment related toxicity*<br>*4 = Other*
[SPCODEATH]	Specify cause of death		[FREE TEXT]

[SEEDMIGR]	Any known seed migration?	|__| *0=no; 1=yes*
[MIGRSPEC]	*If SEEDMIGR=1:* Specify	[FREE TEXT]

EMC 16-400

A MULTICENTER RANDOMIZED CONTROLLED TRIAL TESTING HYALURONIC ACID SPACER INJECTION FOR SKIN TOXICITY REDUCTION OF PERMANENT BREAST SEED IMPLANT (PBSI)

CRF: Primary endpoint assessment (will initially be entered on paper CRF during clinical assessment, and entered in e-CRF later)

Date of assessment		DD / MM / YYYY	
Physician who scored primary endpoint		____	
[TELEANG]	Teleangiectasia (Bentzen)	|__|	*0 =none*<br>*1 = grade I – less than 1cm2*<br>*2 =grade II – 1 to 4 cm2*<br>*3 =grade III – over 4 cm2*
Evaluability of primary endpoint:		|__|	*0=non-evaluable; 1=evaluable*

If non-evaluable, reason: |__| Re-irradiation on ipsilateral breast/chestwall < 2 yrs post-treatment

Re-surgery on ipsilateral breast/chestwall < 2 yrs post treatment

Death < 2 yrs post treatment

Lost to follow-up < 2 yrs post treatment

other, specify

Specify [FREE TEXT]
